# Comparison of the New Viscoelastic Coagulation Analyzer ClotPro® With ROTEM® Delta and Conventional Coagulation Tests in Critically Ill Patients With COVID-19

**DOI:** 10.3389/fmed.2021.777145

**Published:** 2021-11-16

**Authors:** Lukas Infanger, Christoph Dibiasi, Eva Schaden, Stefan Ulbing, Marion Wiegele, Conrad Lacom, Johannes Gratz

**Affiliations:** Department of Anaesthesia, Intensive Care Medicine and Pain Medicine, Medical University of Vienna, Vienna, Austria

**Keywords:** ClotPro®, coagulation, comparison, COVID-19, critically ill, point-of-care, ROTEM Delta®, viscoelastic test

## Abstract

**Background:** Viscoelastic coagulation testing has been suggested to help manage coagulopathy in critically ill patients with COVID-19. However, results from different viscoelastic devices are not readily comparable. ClotPro® is a novel thromboelastometry analyzer offering a wider range of commercially available assays.

**Methods:** We compared the results from ClotPro with results from the well-established ROTEM® Delta device and conventional coagulation tests in critically ill patients with COVID-19.

**Results:** Viscoelastic parameters indicated the presence of a potentially hypercoagulable state in the majority of patients. In up to 95 paired measurements, we found strong correlations between several parameters routinely used in clinical practice: (i) EX test vs. EXTEM CT, A5, A10, MCF, (ii) IN test vs. INTEM A5, A10, MCF, and (iii) FIB test vs. FIBTEM A5, A10, MCF (all *R* > 0.7 and *p* < 0.001). In contrast, IN test CT vs. INTEM CT showed only a moderate correlation (*R* = 0.53 and *p* < 0.001). Clot strength parameters of both devices exhibited strong correlations with platelet counts and fibrinogen levels (all *R* > 0.7 and *p* < 0.001). Divergent correlations of intrinsically activated assays with aPTT and anti-factor Xa activity were visible. Regarding absolute differences of test results, considerable delta occurred in CT, CFT, and clot strength parameters (all *p* < 0.001) between both devices.

**Conclusions:** Several parameters obtained by ClotPro show strong correlations with ROTEM Delta. Due to weak correlations of intrinsically activated clotting times and considerable absolute differences in a number of parameters, our findings underline the need for device-specific algorithms in this patient cohort.

## Introduction

Critically ill patients with COVID-19 might exhibit a number of complex coagulation abnormalities that have been consistently associated with a hypercoagulable state (1). Characteristic findings include the occurrence of endothelial injury (2), increase in factor VIII, von Willebrand factor, and fibrinogen (3), the release of cytokines (4), increased plasma viscosity (5), complement activation (6), and impaired fibrinolysis (7). Conventional coagulation tests such as prothrombin time (PT), activated partial thromboplastin time (aPTT), and antithrombin activity cannot sufficiently depict these changes (8, 9). Recent evidence suggests that viscoelastic testing might be more sensitive to COVID-19-associated coagulopathy in comparison with conventional coagulation tests (10–12). In line with this, a number of recent publications have reported results from viscoelastic tests in patients with COVID-19, including ROTEM (10, 12–16), TEG (17), and ClotPro (1, 7, 18, 19). Additionally, recently published diagnostic pathways for predicting thromboembolic complications and guiding therapeutic interventions in patients with severe COVID-19 suggest that viscoelastic tests be used (11, 12, 15, 20, 21). Moreover, further prospective clinical trials assessing the occurrence of coagulopathy in patients with COVID-19 using viscoelastic tests are underway. However, due to methodological differences, the results of different viscoelastic devices are not interchangeable (22, 23). Studies evaluating the correlations between different viscoelastic coagulation analyzers—such as TEG 5000 and TEG 6s (24, 25), ROTEM Delta and ROTEM Sigma (22, 26), TEG 6s and ROTEM Sigma (27), TEG 5000 and ROTEM Delta (23, 28)—have come up with heterogeneous results, whereas no head-to-head comparison of ClotPro with other viscoelastic point-of-care devices has been published to date.

ClotPro® (enicor GmbH, Munich, Germany) is a novel Conformitè Europëenne (CE) marked viscoelastic whole blood coagulation analyzer that has recently been investigated in patients undergoing orthopedic surgery (29), under treatment with direct oral anticoagulants (30), or with COVID-19 disease (1, 7, 18, 19). In contrast to other viscoelastic devices, ClotPro offers standardized pipette tips prefilled with distinct reagents that allow an ample range of specific assays. Although ClotPro measurements result in thromboelastometry curves similar to those obtained by ROTEM, a direct comparison is pending. Thus, the aim of the present retrospective analysis was to compare simultaneously obtained viscoelastic test results between the novel ClotPro analyzer and the well-established ROTEM® Delta (Tem Innovations GmbH, Munich, Germany) device in critically ill patients with COVID-19.

## Materials and Methods

This retrospective analysis was approved by the Ethics Committee of the Medical University of Vienna (EK 2269/2020) and was performed in accordance with the principles of Good Clinical Practice and the Declaration of Helsinki. We conducted a review of data from all critically ill patients with COVID-19 disease (SARS-CoV2 detected by polymerase chain reaction) admitted to a single tertiary center, the Medical University of Vienna, between 1 April 2020 and 15 December 2020. Two different viscoelastic test systems with a range of diverse assays are regularly used at our institution: ClotPro (enicor GmbH, Munich, Germany, Software Version 1.45a) and ROTEM Delta (Tem Innovations GmbH, Munich, Germany, Software Version 2.8.1). Due to the simultaneous availability of these test systems, viscoelastic tests were run in parallel on both devices in a considerable number of critically ill patients with COVID-19 throughout their ICU stay. Furthermore, all critically ill patients with COVID-19 underwent daily evaluation with conventional coagulation tests at the same timepoint as viscoelastic testing. Blood samples were routinely obtained at the same time every morning by staff of the Intensive Care Units. We thus designed this study to compare results from the novel ClotPro device with (i) results obtained by ROTEM Delta and (ii) conventional coagulation tests. Electronic medical records were reviewed to identify all patients with (i) laboratory-confirmed COVID-19 disease, (ii) admission to an intensive care unit (ICU) and (iii) the presence of simultaneous viscoelastic test results from both devices (i.e., ClotPro and ROTEM) performed from an identical blood sample. Exclusion criteria were (i) pre-existing coagulation disorder, (ii) liver cirrhosis, and (iii) intake of oral anticoagulants within 48 h before admission to the ICU. Patient characteristics, results from viscoelastic tests and laboratory values—of samples drawn at the same time as viscoelastic tests—were extracted into a spreadsheet. Every blood sample with an available viscoelastic test, simultaneously performed on both devices (ClotPro and ROTEM Delta), was included.

ClotPro tests that were routinely performed included (i) EX test [tissue factor (TF)-activated assay), (ii) IN test (ellagic acid-activated assay), (iii) FIB test (TF-activated assay using cytochalasin D and a glycoprotein IIb/IIIa inhibitor for platelet inhibition), and (iv) AP test (TF-activated assay using aprotinin to inhibit fibrinolysis). Corresponding ROTEM Delta tests were (i) EXTEM (TF-activated assay), (ii) INTEM (ellagic acid-activated assay), (iii) FIBTEM (TF-activated assay using cytochalasin D for platelet inhibition), and (iv) APTEM (TF-activated assay using aprotinin to inhibit fibrinolysis). Except for the IN test and INTEM, all tests contain polybrene to neutralize heparin. Measurements and regular quality controls for both viscoelastic devices were run by experienced and trained personnel in accordance with the manufacturers' instructions. We assessed the results of ClotPro and ROTEM Delta and performed a descriptive analysis of the following parameters: coagulation time (CT; time until clot reaches a firmness of 2 mm, measured in s), clot amplitude 5 min after CT (A5; measured in mm), clot amplitude 10 min after CT (A10; measured in mm), clot amplitude 20 min after CT (A20; measured in mm), clot formation time (CFT; time between 2 and 20 mm of clot firmness, measured in s), maximum clot firmness (MCF; maximum amplitude of the clot during measurement, measured in mm), and alpha angle (alpha; angle between baseline and the tangent to the clotting curve through the 2-mm point, measured in degrees). Furthermore, the parameter maximum lysis (ML; measured in percentage of MCF during measurement) was analyzed for tests with an equal runtime of 60 min on both devices. In accordance with clinical routine, blood samples for conventional coagulation tests, platelet count, and viscoelastic testing were drawn from an arterial line at the same time. As CFT of fibrinogen assays is rarely reached in healthy subjects, CFT of FIB test/FIBTEM assays is not used in clinical practice. Furthermore, ClotPro—as set in the device settings by default—did not record results of FIB test CFT, alpha and ML in the database during the study period. Therefore, CFT, alpha, and ML of fibrinogen assays were not included in our analysis.

We analyzed the following conventional coagulation tests: PT (Owren, reference range: 24.6–32.7 s), aPTT (reference range: 27–41 s), fibrinogen (Clauss method; reference range: 2–4 g.l^−1^), and anti-factor Xa activity (STA-Liquid AntiXa, REF 00691 and REF 00322, reference range: <0.1 IU.ml^−1^) via the STA R Max 2 coagulometer (Diagnostica Stago SAS, Asnières-sur-Seine, France). Furthermore, platelet count (reference range: 150–350 10^9^.l^−1^) was determined via the Sysmex XE-2100 cell counter (Sysmex, Kobe, Japan). Normality for continuous data was assessed using the Shapiro–Wilk test. Normally distributed data are presented as mean (SD), whereas non-normally distributed data are presented as median (25–75^th^ percentile). Non-normally distributed variables were compared using the Wilcoxon Signed Rank test. We used Spearman rank correlation to evaluate correlations between the various tests performed on the ClotPro and the ROTEM Delta and standard laboratory parameters. Bland-Altman plots were used to describe agreement between the ClotPro and ROTEM Delta tests. Correlations were considered as very strong correlation (*R* 0.9–1.0), strong correlation (*R* 0.7–0.89), moderate correlation (*R* 0.5–0.69), and weak correlation (*R* 0.3–0.49). We considered two-sided *p*-values ≤ 0.05 to be statistically significant and performed statistical analyses and graphical representations using RStudio (RStudio: Integrated Development for R. RStudio, PBC, Boston, MA, Vers. 4.0.3).

## Results

Between 1 April 2020 and 15 December 2020, 86 critically ill patients with COVID-19 were admitted to an ICU of the Medical University of Vienna. We identified 27 patients (17 men and 10 women) who met the inclusion criteria, resulting in a range of 31 (AP test/APTEM) to 95 (EX test/EXTEM) paired measurements simultaneously performed on ClotPro and ROTEM Delta. Baseline patient characteristics upon ICU admission are depicted in Table 1. Median length of ICU stay was 14 (8–26) days, and throughout their ICU stay, nine (33%) patients received venovenous extracorporeal membrane oxygenation for treatment of acute respiratory distress syndrome. EX test/EXTEM were measured simultaneously at 95 different timepoints in 27 patients, IN test/INTEM at 64 different timepoints in 24 patients, FIB test/FIBTEM at 94 different timepoints in 27 patients, and AP test/APTEM at 31 different timepoints in 16 patients. The median runtime of viscoelastic tests was 60 (60–60) min for ClotPro and 60 (60–60) min for ROTEM Delta. All patients received heparin for pharmacological thromboprophylaxis or anticoagulation. At the time of IN Test/INTEM analysis, 22 (92%) patients received subcutaneous LMWH (Enoxaparin) at a median dose of 80 (40–120) mg.day^−1^, whereas two (8%) patients received continuous intravenous UFH at a mean rate of 1,117 (324) IU.h^−1^. Median time between the last subcutaneous application of LMWH and conduction of IN test/INTEM was 11 (10–14) h. The results of conventional coagulation tests and anti-factor Xa activity at the time of corresponding viscoelastic measurements are depicted in Table 2.

**Table 1:** Patient baseline characteristics upon ICU admission (*n* = 27).

Age (years)tb	58 (15)
Height (cm)	173.8 (7.9)
Weight (kg)	88.8 (16.14)
Male	17 (63%)
BMI (kg.m^−2^)	29.5 (5.8)
SOFA score	11 (10–12)
PaO_2_/FiO_2_ ratio	104 (80–145)
Invasive ventilation	20 (74%)

*Values are mean (SD), median (25–75^th^ percentile), or number (proportion)*.

**Table 2:** Platelet count and conventional coagulation tests at the timepoint of ROTEM/ClotPro measurements.

	**EX test/EXTEM**	**IN test/INTEM**	**FIB test/FIBTEM**
Platelet count (10^9^.l^−1^)	243 (155–353)	235 (154–339)	243 (156–351)
Fibrinogen (g.l^−1^)	6.1 (2)	6.1 (2.2)	6.1 (5–7.1)
PT (s)	30.6 (4.2)	31.0 (4.2)	30.6 (4.2)
INR	1.1 (1.0–1.1)	1.1 (1.0–1.1)	1.1 (1.0–1.1)
aPTT (s)	39 (35–44)	40 (35–45)	39 (35–44)
anti-Xa (IU.ml^−1^)	0.3 (0.2–0.4)	0.2 (0.2–0.3)	0.3 (0.2–0.4)

*Values are mean (SD) and median (25–75^th^ percentile)*.

**Figure 1:**
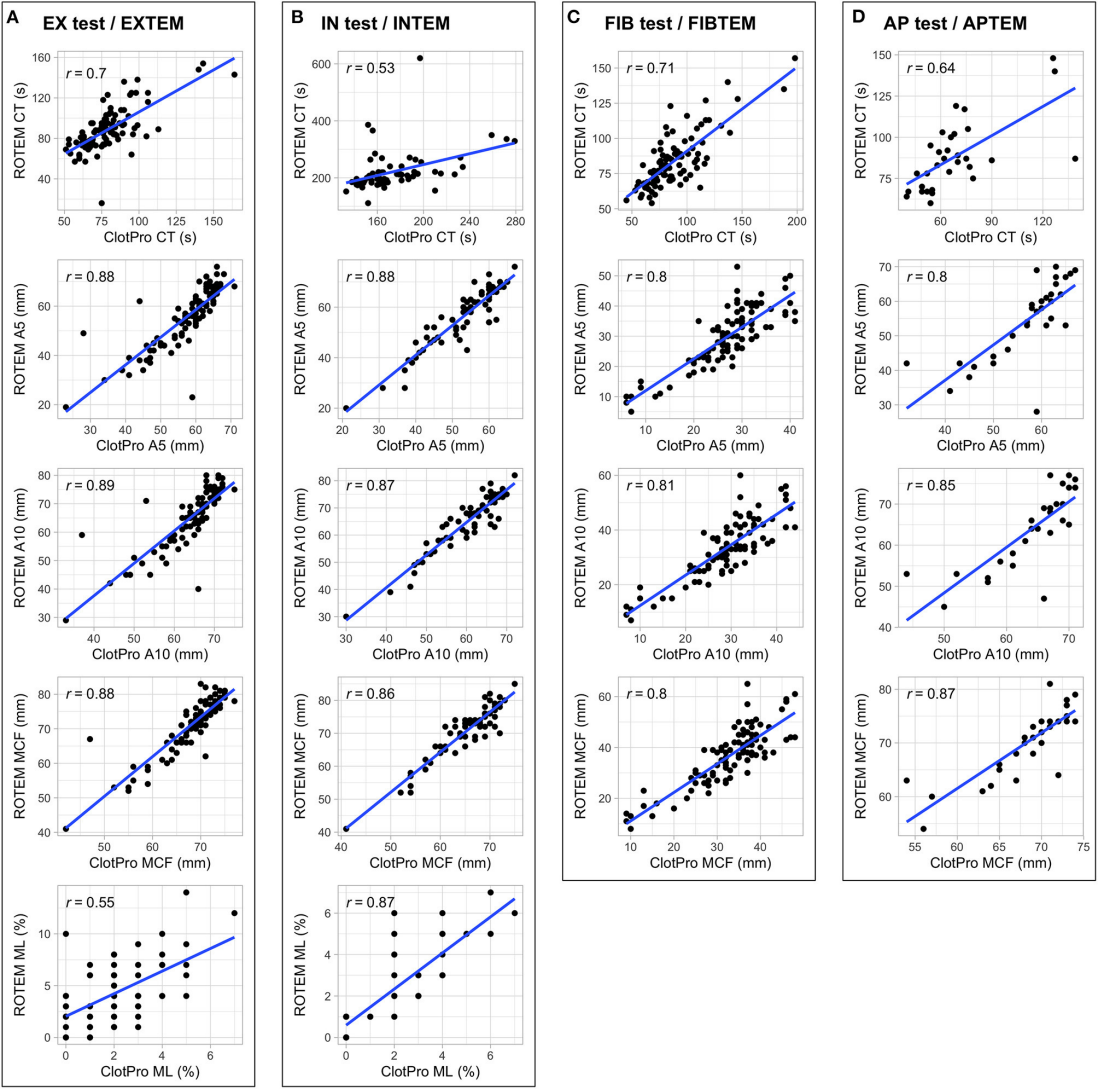
Scatterplots and correlation coefficients for the parameters CT, A5, A10, MCF, and ML for EX test/EXTEM **(A)**, IN test/INTEM **(B)**, FIB test/FIBTEM **(C)**, AP test/APTEM **(D)** performed on both devices.

Figure 1 shows scatterplots and the correlation coefficients for the parameters CT, A5, A10, MCF, and ML for all four distinct assays performed on both devices. We found strong correlations between several parameters routinely used in clinical practice: (i) EX test vs. EXTEM CT, A5, A10, MCF, (ii) IN test vs. INTEM A5, A10, MCF, and (iii) FIB test vs. FIBTEM A5, A10, MCF. In contrast, IN test CT showed only moderate correlations with INTEM CT. Separate analysis for patients receiving LMWH and UFH revealed a similar grade of correlation between IN test CT and INTEM CT (LMWH: 55 timepoints, *r* = 0.45, *p* < 0.001; UFH: 9 timepoints, *r* = 0.47, *p* < 0.001). Scatterplots for further parameters (CFT, alpha, and A20) are shown in Supplementary Figure 1.

Table 3 shows absolute differences (delta) and correlations between ClotPro and ROTEM for EX test/EXTEM, IN test/INTEM, and FIB test/FIBTEM measurements. In decreasing order, considerable absolute differences between the two devices occurred for IN test vs. INTEM CT, EX test vs. EXTEM CT, and EX test vs. EXTEM CFT. Furthermore, clot strength parameters (i.e., A5, A10, A20, and MCF) showed differences, with mainly lower values obtained by ClotPro in extrinsically activated, intrinsically activated, and functional fibrinogen assays. In comparison with ROTEM, ClotPro measurements resulted in smaller interquartile ranges in all assays. Bland-Altmann plots (Supplementary Figure 2) suggested a positive relationship between inter-device differences and the mean of paired measurements for clot strength parameters (i.e., A5, A10, A20, and MCF) throughout all assays. Furthermore, we observed the same pattern for EX test vs. EXTEM ML and IN test vs. INTEM CT. Differences and correlations between AP test/APTEM can be found in Supplementary Table 1.

**Table 3:** Differences and correlations between ROTEM Delta and ClotPro.

	***n***	**Patients**	**ClotPro**	**ROTEM**	Δ^*^	***p*-value^†^**	***r* ^‡^**	***p*-value^‡^**
**EX test/EXTEM**
CT (s)	95	27	77 (68–85)	86 (75–95)	11 (3–18)	<0.001	0.70	<0.001
CFT (s)	95	27	52 (45–76)	60 (49–89)	9 (−4–23)	<0.001	0.60	<0.001
alpha	95	27	78 (75–80)	78 (73–80)	−1 (−3–1)	0.062	0.66	<0.001
A5 (mm)	95	27	60 (54–63)	58 (47–65)	−2 (−6–1)	0.004	0.88	<0.001
A10 (mm)	95	27	66 (62–69)	67 (59–74)	1 (−2–4)	0.028	0.89	<0.001
A20 (mm)	95	27	69 (65–72)	72 (65–77)	3 (0–5)	<0.001	0.89	<0.001
MCF (mm)	95	27	70 (66–72)	73 (68–77)	3 (1–5)	<0.001	0.88	<0.001
ML (%)^§^	69	24	2 (1–3)	4 (2–6)	2 (0–4)	<0.001	0.55	<0.001
**IN test/INTEM**
CT (s)	64	24	165 (155–189)	206 (188–225)	34 (19–54)	<0.001	0.53	<0.001
CFT (s)	64	24	57 (50–80)	62 (50–84)	2 (−4–9)	0.074	0.78	<0.001
alpha	64	24	78 (74–80)	78 (73–80)	0 (−2–1)	0.211	0.79	<0.001
A5 (mm)	64	24	54 (46–60)	58 (48–65)	3 (5)	<0.001	0.88	<0.001
A10 (mm)	64	24	62 (56–66)	67 (59–73)	5 (4)	<0.001	0.87	<0.001
A20 (mm)	64	24	66 (60–69)	72 (66–76)	6 (4)	<0.001	0.86	<0.001
MCF (mm)	64	24	67 (63–70)	72 (68–76)	6 (3)	<0.001	0.86	<0.001
ML (%)^§^	44	21	2 (0–3)	2 (1–4)	0 (0–1)	0.078	0.87	<0.001
**FIB test/FIBTEM**
CT s	95	27	84 (71–100)	81 (70–91)	−1 (−12–4)	0.018	0.71	<0.001
A5 mm	95	27	28 (24–32)	30 (10)	2 (−2–6)	<0.001	0.80	<0.001
A10 mm	95	27	30 (26–34)	34 (11)	4 (0–9)	<0.001	0.81	<0.001
A20 mm	95	27	32 (28–36)	36 (11)	4 (0–10)	<0.001	0.79	<0.001
MCF mm	95	27	35 (29–38)	37 (12)	3 (−1–8)	<0.001	0.80	<0.001

*Values are mean (SD) and median (25–75^th^ percentile). CT, coagulation time; CFT, clot formation time; alpha, alpha angle; A5, clot amplitude 5 min after CT; A10, clot amplitude 10 min after CT; A20, clot amplitude 20 min after CT; MCF, maximum clot firmness; ML, maximum lysis*.

* *Delta*.

† *Wilcoxon signed-rank test*.

‡ *Spearman correlation coefficient*.

§ *Only for tests with a runtime of 60 min*.

**Figure 2:**
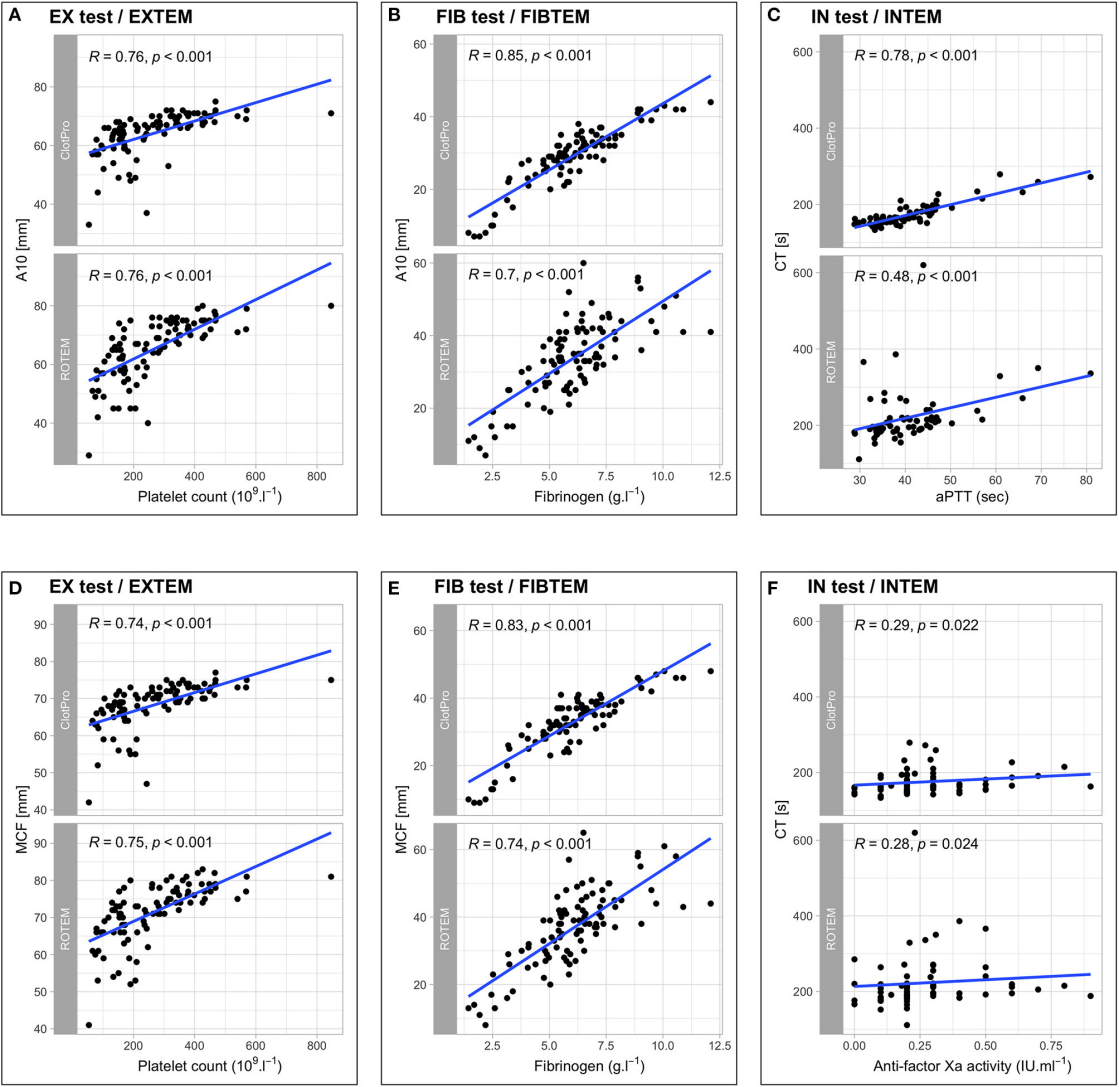
Scatterplots and correlation coefficients between ClotPro/ROTEM measurements and conventional coagulation tests for EX test/EXTEM A10 and platelet count **(A)**, FIB test/FIBTEM A10 and fibrinogen **(B)**, IN test/INTEM CT and aPTT **(C)**, EX test/EXTEM MCF and platelet count **(D)**, FIB test/FIBTEM MCF and fibrinogen **(E)**, IN test/INTEM CT and anti-factor Xa activity **(F)**.

With regard to conventional coagulation tests, we found no correlation between EX test or EXTEM CT and PT (*r* = 0.15, *p* = 0.161 and *r* = 0.16, *p* = 0.132), whereas clot firmness parameters in the extrinsically activated assays (A5, A10, MCF) showed strong correlations with platelet counts. Furthermore, fibrinogen levels correlated strongly with both the FIB test and FIBTEM A10 and MCF. In contrast, intrinsically activated assays revealed differences between the two devices regarding correlations with the aPTT and anti-factor Xa activity. IN test CT showed a strong correlation with aPTT, whereas the correlation between INTEM CT and aPTT was weak. This difference in correlation remained when analyzing for subgroups of pharmacological thromboprophylaxis: LMWH *r* = 0.70, *p* < 0.001 (IN test CT) vs. *r* = 0.40, *p* < 0.001 (INTEM CT); UFH *r* = 0.85, *p* = 0.004 (IN test CT) vs. *r* = 0.38, *p* = 0.308 (INTEM CT). Regarding the correlation between IN test/INTEM CT and anti-factor Xa activity, ClotPro, and ROTEM Delta showed no relevant differences in patients receiving LMWH (*r* = 0.34, *p* = 0.012 vs. *r* = 0.36, *p* = 0.007, respectively), whereas different correlations occurred in patients receiving UFH (*r* = 0.75, *p* = 0.020 vs. *r* = 0.55, *p* = 0.125, respectively). Scatterplots and correlation coefficients between ClotPro/ROTEM measurements and conventional coagulation tests are depicted in Figure 2.

## Discussion

To the best of our knowledge, we are the first to present a direct head-to-head comparison between the novel ClotPro analyzer and the well-established ROTEM Delta device in a clinical setting, i.e., a cohort of critically ill patients with COVID-19. Critically ill patients with COVID-19 regularly exhibit hemostatic alterations associated with an increased risk for thromboembolic complications (31), and conventional coagulation tests fail to adequately depict these complex changes (9). Hence, a multimodal diagnostic approach, including the use of viscoelastic testing, may confer benefits in the management of this specific patient cohort. However, the comparability of results obtained by different viscoelastic devices has repeatedly been questioned due to methodological discrepancies (22, 23, 28). Therefore, the assessment of correlation between results from distinct viscoelastic devices in defined patient populations is crucial.

Generally, the characteristics of patients included in the present study were similar in comparison to current literature describing the use of viscoelastic tests in critically ill patients with COVID-19. Our patients showed a higher sequential organ failure assessment (SOFA) score, a lower Horowitz index and younger age (1, 7, 10, 12, 13, 15, 32). This can be explained by the fact that the majority of patients were transferred from other hospitals to our ICUs for extracorporeal life support evaluation. Regarding conventional coagulation tests, our results corroborate those found in current literature, largely reporting high levels of fibrinogen, normal to increased platelet counts, and normal or slightly prolonged PT and aPTT (1, 7, 14, 15, 32, 33). Moreover, our viscoelastic test results are comparable with those reported by other study groups (1, 7, 11, 12, 32, 34). In line with a possible shift of haemostatic balance toward a prothrombotic state in critically ill patients with COVID-19, the median results of most viscoelastic clot strength parameters exceeded both manufacturer reference ranges and values of healthy controls (1, 7, 35).

Critical illness due to COVID-19 has been shown to result in similarly high rates of venous thromboembolism compared to critically ill patients with sepsis (36). Both patient cohorts share a number of similar pathophysiologic features, such as the excessive release of cytokines. In this regard, impaired fibrinolysis has been previously described as a hallmark of both sepsis-induced and COVID-19-associated coagulopathy (7, 10, 37). Of particular interest, impaired fibrinolysis cannot be depicted by conventional coagulation tests. In our study, fibrinolytic shutdown, previously defined as an ML <3.5% in patients with sepsis and severe trauma (37, 38), was present in 26 (96%) of the included patients at some timepoint throughout their ICU stay (extrinsic TF-activated assay; ClotPro or ROTEM Delta). Notably, however, ClotPro might provide a higher sensitivity with regard to detecting impaired fibrinolysis: 57 (82%) of ClotPro EX test results fulfilled the criteria for fibrinolytic shutdown, whereas only 32 (46%) of ROTEM Delta EXTEM results did so. These differences might be of importance when designing viscoelastically guided algorithms for the treatment of this specific patient cohort.

Patients included in the present study received heparin for pharmacological thromboprophylaxis or therapeutic anticoagulation. Despite the use of mainly intermediate doses of LMWH and UFH, we found both IN test and INTEM CT values to remain within normal values. The manufacturers of ClotPro and ROTEM provide divergent reference ranges: IN test CT 100–240 s and INTEM CT 136–187 s, which is in line with available published results of healthy controls (Bachler et al.: IN test CT 153–166 s; Lang et al.: INTEM CT 137–246 s) (7, 35). These differences might be attributable to distinct reagent compositions and might explain the poor correlation between the IN test and INTEM CT found in our study. Up to date, no evidence-based viscoelastically guided algorithms for patients with COVID-19 have been established. But literature suggesting the use of viscoelastic testing to guide prevention and treatment of thromboembolic complications in this patient cohort has emerged (39, 40). Against this background, our findings represent a clinically relevant discovery. In clinical practice, the moderate correlation of IN test/INTEM CT implies that algorithms based on results from one device cannot readily be used with results obtained from the other device. On the other hand, some statistically significant absolute differences between these two devices might not translate into clinical relevance. For example, a difference between FIB test and FIBTEM CT would almost certainly not result in changes in patient management, whereas different results of clot strength parameters (A5, A10, MCF) might lead to alterations in medical interventions in bleeding patients (41, 42).

Regarding conventional coagulation tests, we found correlations between (i) EX test/EXTEM MCF and platelet count and (ii) FIB test/FIBTEM MCF and fibrinogen that were stronger than previously reported (19). Furthermore, we found strong correlations between IN test CT and aPTT as well as anti-factor Xa activity, except for anti-Xa levels obtained in patients receiving LMWH. In contrast, INTEM CT showed moderate to no correlations with aPTT and anti-Xa levels. A previous study found moderate (*r* = 0.69, *p* < 0.001) correlations between ROTEM INTEM CT and aPTT in patients not receiving heparin and healthy volunteers, whereas in our study population ROTEM INTEM CT only showed poor correlations in comparison with aPTT (38). Our findings suggest that the novel ClotPro might show higher sensitivity for UFH than ROTEM Delta.

The limitations of the present study need to be recognized. First, the retrospective design of our study carries an inherent risk of bias. Second, baseline characteristics indicate a selection of patients that were most severely affected by COVID-19, which can be explained by the status of our institution as an ECMO referral center. Finally, our study reports results obtained from a rather small sample size. Taken together with the fact that we conducted a single-center study, this might limit the generalizability and transferability of our results. Although we are convinced that our findings add important knowledge to the field of viscoelastic testing in critically ill patients, they remain to be confirmed by future prospective multi-center trials.

In conclusion, we found several parameters obtained by the novel viscoelastic coagulation analyzer ClotPro to show strong correlations with the well-established ROTEM Delta device in critically ill patients with COVID-19. In contrast, intrinsically activated clotting times showed only weak correlations. Moreover, in a large number of parameters, considerable differences occurred between the results from the two devices. Although these results remain to be confirmed by prospective trials, our findings underline the need for the development of device-specific algorithms in defined patient cohorts.

## Data Availability Statement

The raw data supporting the conclusions of this article will be made available by the authors, without undue reservation.

## Ethics Statement

The studies involving human participants were reviewed and approved by Ethics Committee of the Medical University of Vienna. Written informed consent for participation was not required for this study in accordance with the national legislation and the institutional requirements.

## Author Contributions

LI and JG: study conception and design and first draft of the manuscript. LI, CD, ES, SU, MW, CL, and JG: data collection and interpretation. LI and CD: statistical analysis. All authors commented on previous versions of the manuscript. All authors read and approved the final manuscript.

## Funding

This work was supported by departmental funds of the Department of Anaesthesia, Critical Care and Pain Medicine, Division of General Anaesthesia and Intensive Care Medicine, Medical University of Vienna.

## Conflict of Interest

MW received speaker's fees from Tem Innovations GmbH and Enicor GmbH. JG has received honoraria, research funding, and travel reimbursement from Alexion, Boehringer Ingelheim, CSL Behring, Johnson & Johnson, Instrumentation Laboratory, Mitsubishi Tanabe Pharma, Octapharma, and Portola. The remaining authors declare that the research was conducted in the absence of any commercial or financial relationships that could be construed as a potential conflict of interest.

## Publisher's Note

All claims expressed in this article are solely those of the authors and do not necessarily represent those of their affiliated organizations, or those of the publisher, the editors and the reviewers. Any product that may be evaluated in this article, or claim that may be made by its manufacturer, is not guaranteed or endorsed by the publisher.

## References

[1] Heinz C Miesbach W Herrmann E Sonntagbauer M Raimann FJ Zacharowski K. Greater fibrinolysis resistance but no greater platelet aggregation in critically ill COVID-19 patients. Anesthesiology. (2021) 134:457–67. 10.1097/ALN.0000000000003685 33417674 PMC7864605

[2] Lowenstein CJ Solomon SD. Severe COVID-19 is a microvascular disease. Circulation. (2020) 142:1609–11. 10.1161/CIRCULATIONAHA.120.050354 32877231 PMC7580651

[3] Panigada M Bottino N Tagliabue P Grasselli G Novembrino C Chantarangkul V. Hypercoagulability of COVID-19 patients in intensive care unit: a report of thromboelastography findings and other parameters of hemostasis. J Thromb Haemost. (2020) 18:1738–42. 10.1111/jth.14850 32302438 PMC9906150

[4] Begbie M Notley C Tinlin S Sawyer L Lillicrap D. The factor VIII acute phase response requires the participation of NFkappaB and C/EBP. Thromb Haemost. (2000) 84:216–22. 10.1055/s-0037-1613999 10959692

[5] Maier CL Truong AD Auld SC Polly DM Tanksley C-L Duncan A. COVID-19-associated hyperviscosity: a link between inflammation and thrombophilia? Lancet. (2020) 395:1758–9. 10.1016/S0140-6736(20)31209-5 32464112 PMC7247793

[6] Ma L Sahu SK Cano M Kuppuswamy V Bajwa J McPhatter JN. Increased complement activation is a distinctive feature of severe SARS-CoV-2 infection. Sci Immunol. (2021) 6:eabh2259. 10.1126/sciimmunol.abh2259 34446527 PMC8158979

[7] Bachler M Bosch J Sturzel DP Hell T Giebl A Strohle M. Impaired fibrinolysis in critically ill COVID-19 patients. Br J Anaesth. (2021) 126:590–8. 10.1016/j.bja.2020.12.010 33422287 PMC7833514

[8] Helms J Tacquard C Severac F Leonard-Lorant I Ohana M Delabranche X. High risk of thrombosis in patients with severe SARS-CoV-2 infection: a multicenter prospective cohort study. Intensive Care Med. (2020) 46:1089–98. 10.1007/s00134-020-06062-x 32367170 PMC7197634

[9] Mortus JR Manek SE Brubaker LS Loor M Cruz MA Trautner BW. Thromboelastographic results and hypercoagulability syndrome in patients with coronavirus disease 2019 who are critically ill. JAMA Netw Open. (2020) 3:e2011192. 10.1001/jamanetworkopen.2020.11192 32501489 PMC7275245

[10] Creel-Bulos C Auld SC Caridi-Scheible M Barker NA Friend S Gaddh M. Fibrinolysis shutdown and thrombosis in a COVID-19 ICU. Shock (Augusta, Ga). (2021) 55:316–20. 10.1097/SHK.0000000000001635 32769822 PMC8858425

[11] Wright FL Vogler TO Moore EE Moore HB Wohlauer MV Urban S. Fibrinolysis shutdown correlation with thromboembolic events in severe COVID-19 infection. J Am Coll Surg. (2020) 231:193.e1–203.e1. 10.1016/j.jamcollsurg.2020.05.007 32422349 PMC7227511

[12] Kruse JM Magomedov A Kurreck A Munch FH Koerner R Kamhieh-Milz J. Thromboembolic complications in critically ill COVID-19 patients are associated with impaired fibrinolysis. Crit Care. (2020) 24:676. 10.1186/s13054-020-03401-8 33287877 PMC7719734

[13] Nougier C Benoit R Simon M Desmurs-Clavel H Marcotte G Argaud L. Hypofibrinolytic state and high thrombin generation may play a major role in SARS-COV2 associated thrombosis. J Thromb Haemost. (2020) 18:2215–9. 10.1111/jth.15016 32668058 PMC7405476

[14] van Veenendaal N Scheeren TWL Meijer K van der Voort PHJ. Rotational thromboelastometry to assess hypercoagulability in COVID-19 patients. Thromb Res. (2020) 196:379–81. 10.1016/j.thromres.2020.08.046 32980621 PMC7462575

[15] Almskog LM Wikman A Svensson J Wanecek M Bottai M van der Linden J. Rotational thromboelastometry results are associated with care level in COVID-19. J Thromb Thrombolysis. (2021) 51:437–45. 10.1007/s11239-020-02312-3 33068277 PMC7568025

[16] Weiss E Roux O Moyer JD Paugam-Burtz C Boudaoud L Ajzenberg N. Fibrinolysis resistance: a potential mechanism underlying COVID-19 coagulopathy. Thromb Haemost. (2020) 120:1343–5. 10.1055/s-0040-1713637 32645725

[17] Maatman TK Jalali F Feizpour C Douglas A II McGuire SP Kinnaman G. Routine venous thromboembolism prophylaxis may be inadequate in the hypercoagulable state of severe coronavirus disease 2019. Crit Care Med. (2020) 48:e783–90. 10.1097/CCM.0000000000004466 32459672 PMC7302085

[18] Duque P Chasco-Ganuza M Burgos-Santamaria A Terradillos E. Increased resistance to fibrinolysis activation in patients with coronavirus disease 2019: a case series. Blood Coagul Fibrinolysis. (2021) 32:298–301. 10.1097/MBC.0000000000001017 33491994

[19] Mitrovic M Sabljic N Cvetkovic Z Pantic N Zivkovic Dakic A Bukumiric Z. Rotational thromboelastometry (ROTEM) profiling of COVID-19 patients. Platelets. (2021) 32:690–6. 10.1080/09537104.2021.1881949 33561381

[20] Kichloo A Dettloff K Aljadah M Albosta M Jamal S Singh J. COVID-19 and hypercoagulability: a review. Clin Appl Thromb Hemost. (2020) 26:1076029620962853. 10.1177/1076029620962853 33074732 PMC7592310

[21] Wang J Hajizadeh N Moore EE McIntyre RC Moore PK Veress LA. Tissue plasminogen activator (tPA) treatment for COVID-19 associated acute respiratory distress syndrome (ARDS): a case series. J Thromb Haemost. (2020) 18:1752–5. 10.1111/jth.14828 32267998 PMC7262152

[22] Gillissen A van den Akker T Caram-Deelder C Henriquez D Bloemenkamp KWM Eikenboom J. Comparison of thromboelastometry by ROTEM((R)) delta and ROTEM((R)) sigma in women with postpartum haemorrhage. Scand J Clin Lab Invest. (2019) 79:32–8. 10.1080/00365513.2019.1571220 30727759

[23] Hagemo JS Naess PA Johansson P Windelov NA Cohen MJ Roislien J. Evaluation of TEG((R)) and RoTEM((R)) inter-changeability in trauma patients. Injury. (2013) 44:600–5. 10.1016/j.injury.2012.11.016 23260867

[24] Gurbel PA Bliden KP Tantry US Monroe AL Muresan AA Brunner NE. First report of the point-of-care TEG: a technical validation study of the TEG-6S system. Platelets. (2016) 27:642–9. 10.3109/09537104.2016.1153617 27809712

[25] Neal MD Moore EE Walsh M Thomas S Callcut RA Kornblith LZ. A comparison between the TEG 6s and TEG 5000 analyzers to assess coagulation in trauma patients. J Trauma Acute Care Surg. (2020) 88:279–85. 10.1097/TA.0000000000002545 31738314 PMC7004476

[26] Schenk B Gorlinger K Treml B Tauber H Fries D Niederwanger C. A comparison of the new ROTEM((R)) sigma with its predecessor, the ROTEMdelta. Anaesthesia. (2019) 74:348–56. 10.1111/anae.14542 30575011

[27] Ziegler B Voelckel W Zipperle J Grottke O Schochl H. Comparison between the new fully automated viscoelastic coagulation analysers TEG 6s and ROTEM Sigma in trauma patients: a prospective observational study. Eur J Anaesthesiol. (2019) 36:834–42. 10.1097/EJA.0000000000001032 31219873

[28] Solomon C Sorensen B Hochleitner G Kashuk J Ranucci M Schochl H. Comparison of whole blood fibrin-based clot tests in thrombelastography and thromboelastometry. Anesth Analg. (2012) 114:721–30. 10.1213/ANE.0b013e31824724c8 22314689

[29] Groene P Sappel SR Saller T Nitschke T Sa PA Paulus A. Functional testing of tranexamic acid effects in patients undergoing elective orthopaedic surgery. J Thromb Thrombolysis. (2020) 51:989–96. 10.1007/s11239-020-02272-8 32918670

[30] Oberladstatter D Voelckel W Schlimp C Zipperle J Ziegler B Grottke O. A prospective observational study of the rapid detection of clinically-relevant plasma direct oral anticoagulant levels following acute traumatic injury. Anaesthesia. (2020) 76:373–80. 10.1111/anae.15254 32946123

[31] Bilaloglu S Aphinyanaphongs Y Jones S Iturrate E Hochman J Berger JS. Thrombosis in hospitalized patients with COVID-19 in a New York City health system. JAMA. (2020) 324:799–801. 10.1001/jama.2020.13372 32702090 PMC7372509

[32] Tsantes AE Frantzeskaki F Tsantes AG Rapti E Rizos M Kokoris SI. The haemostatic profile in critically ill COVID-19 patients receiving therapeutic anticoagulant therapy: an observational study. Medicine (Baltimore). (2020) 99:e23365. 10.1097/MD.0000000000023365 33217881 PMC7676559

[33] Ranucci M Ballotta A Di Dedda U Bayshnikova E Dei Poli M Resta M. The procoagulant pattern of patients with COVID-19 acute respiratory distress syndrome. J Thromb Haemost. (2020) 18:1747–51. 10.1111/jth.14854 32302448 PMC9906332

[34] Correa TD Cordioli RL Campos Guerra JC Caldin da. Silva B, Dos Reis Rodrigues R, de Souza GM, et al. Coagulation profile of COVID-19 patients admitted to the ICU: an exploratory study. PLoS ONE. (2020) 15:e0243604. 10.1371/journal.pone.0243604 33320874 PMC7737963

[35] Lang T Bauters A Braun SL Potzsch B von Pape KW Kolde HJ. Multi-centre investigation on reference ranges for ROTEM thromboelastometry. Blood Coagul Fibrinolysis. (2005) 16:301–10. 10.1097/01.mbc.0000169225.31173.19 15870552

[36] Gratz J Wiegele M Maleczek M Herkner H Schochl H Chwala E. Risk of clinically relevant venous thromboembolism in critically ill patients with COVID-19: a systematic review and meta-analysis. Front Med (Lausanne). (2021) 8:647917. 10.3389/fmed.2021.647917 33768106 PMC7985162

[37] Adamzik M Eggmann M Frey UH Gorlinger K Brocker-Preuss M Marggraf G. Comparison of thromboelastometry with procalcitonin, interleukin 6, and C-reactive protein as diagnostic tests for severe sepsis in critically ill adults. Crit Care. (2010) 14:R178. 10.1186/cc9284 20929576 PMC3219282

[38] Scala E Coutaz C Gomez F Alberio L Marcucci C. Comparison of ROTEM sigma to standard laboratory tests and development of an algorithm for the management of coagulopathic bleeding in a tertiary center. J Cardiothorac Vasc Anesth. (2020) 34:640–9. 10.1053/j.jvca.2019.10.016 31699598

[39] Bunch CM Thomas AV Stillson JE Gillespie L Khan RZ Zackariya N. Preventing thrombohemorrhagic complications of heparinized COVID-19 patients using adjunctive thromboelastography: a retrospective study. J Clin Med. (2021) 10:3097. 10.3390/jcm10143097 34300263 PMC8303660

[40] Seelhammer TG Plack D Lal A Nabzdyk CGS. COVID-19 and ECMO: an unhappy marriage of endothelial dysfunction and hemostatic derangements. J Cardiothorac Vasc Anesth. (2020) 34:3193–6. 10.1053/j.jvca.2020.09.132 33228917 PMC7531342

[41] Kozek-Langenecker SA Ahmed AB Afshari A Albaladejo P Aldecoa C Barauskas G. Management of severe perioperative bleeding: guidelines from the European Society of Anaesthesiology: first update 2016. Eur J Anaesthesiol. (2017) 34:332–95. 10.1097/EJA.0000000000000630 28459785

[42] Curry NS Davenport R Pavord S Mallett SV Kitchen D Klein AA. The use of viscoelastic haemostatic assays in the management of major bleeding: a British Society for Haematology Guideline. Br J Haematol. (2018) 182:789–806. 10.1111/bjh.15524 30073664

