# Supplementary material for: Comparison of the New Viscoelastic Coagulation Analyzer ClotPro® With ROTEM® Delta and Conventional Coagulation Tests in Critically Ill Patients With COVID-19
Source: Front Med (Lausanne). 2021 Nov 16;8:777145. doi: 10.3389/fmed.2021.777145 (PMC8635118; doi:10.3389/fmed.2021.777145)
Supplement: Supplementary file 4 [file Table_1.DOCX]

***Supplementary Table 1****: Differences and correlations between ROTEM Delta APTEM and ClotPro AP test. Values are mean (SD) and median median (25 – 75^th^ percentile).*

| AP test / APTEM | | | | | | | | |
| --- | --- | --- | --- | --- | --- | --- | --- | --- |
|  | n | Patients | ClotPro | ROTEM | ∆ * | p Value† | r‡ | p Value‡ |
| CT (s) | 31 | 16 | 64 (54 - 75) | 86 (70 – 100) | 22 (13 - 32) | <0.001 | 0.64 | <0.001 |
| CFT (s) | 31 | 16 | 67 (52 - 77) | 67 (53 - 96) | 3 (-7 - 12) | 0.382 | 0.53 | 0.020 |
| alpha | 31 | 16 | 78 (76 - 80) | 77 (73 – 80) | -1 (-3 - 0) | 0.30 | 0.60 | <0.001 |
| A5 (mm) | 31 | 16 | 59 (53 - 63) | 54 (11) | -2 (-7 - 2) | 0.052 | 0.80 | <0.001 |
| A10 (mm) | 31 | 16 | 67 (61 - 69) | 64 (9) | 1 (-3 – 3) | 0.837 | 0.85 | <0.001 |
| A20 (mm) | 31 | 16 | 69 (65 - 72) | 69 (7) | 2 (-1 – 4) | 0.031 | 0.82 | <0.001 |
| MCF (mm) | 31 | 16 | 70 (67 - 73) | 70 (6) | 2 (0 – 4) | 0.004 | 0.87 | <0.001 |
| * Delta | | | | |  |  |  |  |
| † Wilcoxon signed-rank test | | | | |  |  |  |  |
| ‡ Spearman correlation coefficient  CT, coagulation time; CFT, clot formation time; alpha, alpha angle; A5, clot amplitude 5 min after CT; A10, clot amplitude 10 min after CT; A20, clot amplitude 20 min after CT; MCF, maximum clot firmness; ML, maximum lysis; | | | | | | | | |
